# Nodal status in luminal A invasive breast cancer: relationships with cytotoxic CD8 + and regulatory FOXP3 + cells tumor-associated infiltrate and other prognostic factors

**DOI:** 10.1007/s00428-021-03126-1

**Published:** 2021-06-12

**Authors:** Anna Glajcar, Agnieszka Łazarczyk, Katarzyna Ewa Tyrak, Diana Hodorowicz-Zaniewska, Joanna Streb, Krzysztof Okoń, Joanna Szpor

**Affiliations:** 1 Department of Pathomorphology, Jagiellonian University Medical College, Cracow, Poland; 2 Department of Pathomorphology, University Hospital, Cracow, Poland; 3 2Nd Department of Internal Medicine, Jagiellonian University Medical College, Cracow, Poland; 4 I Chair of General, Oncological, and Gastrointestinal Surgery, Jagiellonian University Medical College, Cracow, Poland; 5 University Center for Breast Diseases, University Hospital, Cracow, Poland; 6 Department of Oncology, Jagiellonian University Medical College, Cracow, Poland

**Keywords:** Breast cancer, Luminal A, Cytotoxic cells, Regulatory cells, Nodal stage

## Abstract

Luminal A breast cancers are generally associated with low metastatic potential and good prognosis. However, there is a proportion of patients, who present with metastases in lymph nodes. The aim of our study was to determine the association between the number of positive lymph nodes and infiltrates of tumor-associated cytotoxic CD8 + (CTLs), regulatory FOXP3 + T cells (Tregs), as well as other prognostic factors. Immunohistochemistry (IHC) for CD8 + and FOXP3 + was performed in 87 formalin-fixed paraffin-embedded primary breast cancer tissues, and cell infiltrate was assessed under light microscope. We observed that node-positive cases were associated with higher numbers of Treg cells and lower CTL/Treg ratio. There was also an inverse correlation between the CTL/Treg ratio and the number of metastatic lymph nodes. Similar relationships were found between the number of metastatic lymph nodes and Treg density or CTL/Treg ratio in pT1 BC. An elevated intratumoral CTL/Treg ratio was associated with pN0 stage. The relationship between lymphovascular invasion (LVI) and Treg density was also noted in node-negative tumors. In addition, more advanced nodal stage was related to LVI, higher pT, and lower PR expression. The numbers of CD8 + and FOXP3 + were also associated with tumor size, histologic grade, PR expression, and mitotic index. The results of our study suggested that the levels of tumor-infiltrating regulatory and cytotoxic cells as well as the balance between them play a role in lymphovascular spread of luminal A breast cancers.

## Introduction

Breast cancer (BC) is the most common cancer in women in both developed and developing countries. In 2018, 2.1 million new cases were diagnosed worldwide, with mortality of over 600,000 women [1]. The incidence of BC is still rising, which is caused by the increase in world population and longer life expectancy [2]. The presence of cancerous tissue in lymph nodes is associated with worse prognosis. It is an important element of staging, used to assess patient prognosis and to choose the appropriate treatment method. Lymph node metastases reflect interaction between the tumor and the immune system and also promote the spread of cancer cells to distant sites of the patient’s body [3].

In clinical practice, the most commonly used molecular classification of BC is St Gallen 2015 International Expert Consensus, based on the expression of an estrogen receptor (ER), progesterone receptor (PR), a receptor for human epidermal growth factor 2 (HER2) in cancer cells and Ki67 protein, which reflects proliferation levels [4, 5]. Currently, five molecular types of BC are distinguished: luminal type A (ER + , PR + , HER2-, low Ki67), HER2- luminal type B (ER + , low PR or high Ki67, HER2-), luminal B/HER2 + (ER + and HER2 +), HER2-positive non-luminal (ER-, PR-, HER2 +). and triple-negative BC (TNBC; ER-, PR-, HER2-) [6]. Immunohistochemistry (IHC) is a technique routinely used to assess BC subtype [4, 5]. The cell phenotype defined this way is an important parameter while choosing treatment and assessing prognosis.

Luminal A is the most common molecular subtype of BC, accounting for up to 60–70% of cases. It is characterized by the most favorable prognosis, resulting from its positive response to hormonal treatment and low metastatic potential. However, a tendency to cancer spread in its later stages (often more than 5 years after the onset of symptoms) is observed [6, 7], and metastases are mostly found in lymph nodes and bones [8].

The tumor microenvironment consists of elements in the close vicinity of cancer cells, such as extracellular matrix, fibroblasts, blood and lymphatic vessels, as well as immune cells (including various lymphocyte subpopulations). As in luminal A, the number of tumor-infiltrating lymphocytes (TILs) is relatively small [9]; this subtype of BC is regarded as less immunogenic than the other [6]. The presence of TILs is regarded as a favorable prognostic factor, in particular if they are dominated by CD8^+^ cytotoxic lymphocytes (CTLs) [10]. They exert their anti-tumor properties primarily by the induction of apoptosis in cancer cells and the decrease in their proliferation rate. Through the production of interferon (IFNγ), CTLs stimulate the transformation of macrophages to M1 type, a phenotype with anti-cancer properties [11]. On the contrary, the presence of regulatory T lymphocytes (Tregs), characterized by the expression of the Forkhead box P3 (FoxP3) transcription factor, provides tolerance to tumor antigens, thus promotes its development [12] and worsens patient prognosis [13]. In BC with nodal metastases, a higher number of Tregs was found in primary tumors, but this association was not clearly determined for luminal A cancer [14, 15].

The aim of our study was to determine whether the infiltrate of CD8^+^ CTLs and FOXP3^+^ Tregs in the microenvironment of primary invasive luminal A BC is associated with lymph node involvement or other prognostic factors. We believe this would provide missing information about the impact of tumor microenvironment on the occurrence of lymph node metastases and the progression of luminal A tumors. Additionally, we investigated the relationships between nodal stage and other prognostic factors in this type of BC.

## Materials and methods

### Material

The material comprised 87 routinely processed, formalin-fixed paraffin-embedded tissues of primary invasive BC diagnosed between 2011 and 2018. All cases were classified as luminal A molecular subtypes according to St Gallen 2015 International Expert Consensus [16]: ER + , PR ≥ 20%, Ki67 < 20%, and HER2-. The tissue material from patients who received presurgical chemotherapy was excluded from the study. The archival hematoxylin–eosin-stained slides were re-evaluated, and representative, well-preserved specimens were chosen for immunohistochemistry. The information on numbers of metastatic lymph nodes and the presence of lymphovascular invasion was obtained by reviewing medical records. Nottingham Histologic Grade system was used for grading, and the eighth edition of AJCC system was used for staging [17].

### Immunohistochemistry

IHC for CD8, FOXP3, ER, PR, and Ki67 was performed according to the protocol routinely used in our laboratory. The selected blocks were cut into 4-μm-thick sections. Antigen retrieval was performed by incubating the slides in citrate buffer (pH 6.0; 0.01 M) or EDTA (pH 8.0; 0.01 M) at 97 °C in a water bath for 40 and 30 min, respectively. UltraVision Quanto detection system (Lab Vision, ThermoScientific, USA) and 3,3’-diaminobenzidine as chromogen were used, and the slides were counterstained with Mayer hematoxylin (Thermo Fisher Scientific, Waltham, USA), and coverslipped. IHC for HER2 (PATHWAY 4B5, Ventana Medical Systems Inc., USA) was performed on BenchMark BMK Classic autostainer (Ventana, USA) using UltraVIEW DAB Detection Kit (Ventana Medical Systems Inc., USA). Primary antibodies used are listed in Table 1.

**Table 1:** Antibodies used in the study

	Clone	Dilution	Antigen retrieval	Incubation time	Manufacturer
CD8	C8/144B	1:100	Citrate	60 min	Dako, USA
FOXP3	236A/E7	1:100	EDTA	30 min	Abcam, UK
ER	6F11	1:100	Citrate	30 min	Novocastra (Leica Biosystems, Germany)
PR	PgR636	1:100	Citrate	60 min	Dako, USA
Ki67	MIB-1	1:100	Citrate	30 min	Dako, USA

For specimens with HER2 status 2 + in IHC, fluorescence in situ hybridization (FISH) was conducted. FISH was performed using a PathVysion HER-2 DNA Probe Kit II (Abbott Molecular, USA) according to the manufacturer’s protocol. The LSI HER-2/neu and CEP17 signals were counted on fluorescence microscope equipped with specific filter sets, and HER-2/neu to CEP17 ratio > 2.0 was considered as HER2/neu overexpression [18].

### Evaluation of immunostaining

Positive ER and PR expression thresholds were set when ≥ 1% of neoplastic cells showed positive immunostaining. The threshold for discriminating between low and high Ki67 expression was set at ≥ 20% of positive cells. Scoring of the HER2 staining was performed by standard method [18].

### Evaluation of lymphocytic infiltrate

**Fig. 1:**
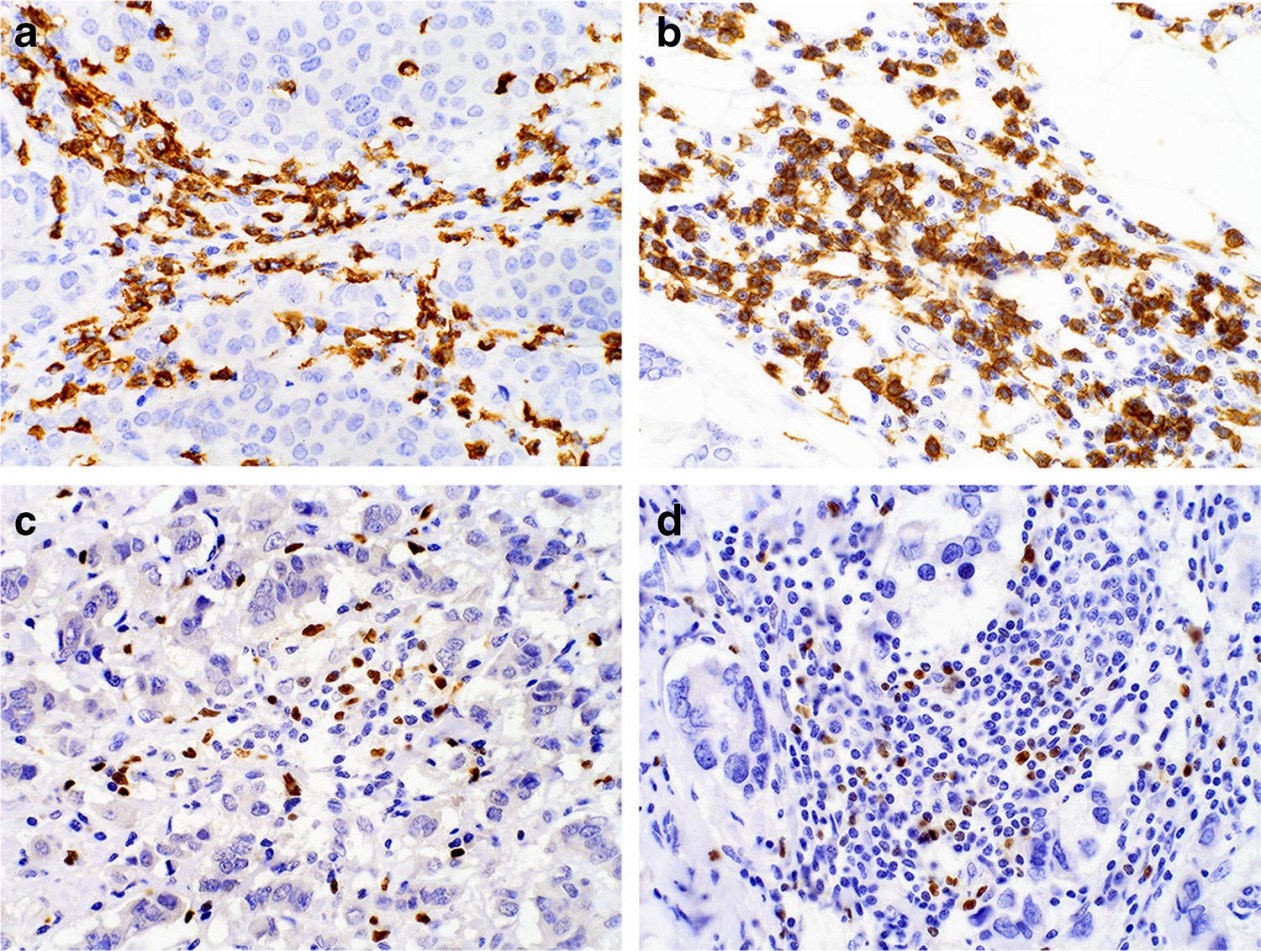
The exemplary microphotographs of investigated immune cell populations in BC tissue: **A** Intratumoral CD8 + CTLs (CTLs number = 149), **B** invasive margin CD8 + CTLs (CTLs number = 231, CTL percentage = 55.8%), **C** intratumoral FOXP3 + Tregs (Tregs number = 54), **D** invasive margin FOXP3 + Tregs (Tregs number = 62, Treg percentage = 13.3%). Magnification 400 ×

The immunostained slides were initially scanned on Olympus BX53 optical microscope (Olympus Corporation, Tokyo, Japan) at low magnification (100 ×), and the areas with the highest number of CD8-positive (CTLs) or FOXP3-positive (Tregs) cells were chosen. Then, digital microphotographs of 5 high-power fields (HPFs; 400 ×) in non-overlapping areas were taken using Olympus SC180 camera (Olympus Corporation, Tokyo, Japan). Positively stained CTLs and Tregs were counted in the microphotographs with the use of Olympus CellSens Standard 2.3 software (Olympus Corporation, Tokyo, Japan) and its tool object counting. The positive cells located in tumor surrounding stroma, no further than 1 HPF from the tumor edge, were regarded as invasive margin, while positive cells located within cancerous tissue were considered as intratumoral population (Fig. 1). In detail, in each microphotograph, two populations of cells (positively stained cells and cells lacking CD8 or FOXP3 expression) were labelled separately in mononuclear infiltrate located at invasive margin. The sum of the two labelled cell populations obtained in each slide represented the total mononuclear infiltrate. The numbers of positively stained cells (CTL or Treg) as well as their percentages in mononuclear infiltrate obtained in each image were calculated automatically by the software. For intratumoral population, only positively stained cells were labelled, and their numbers were noted. Cell counts obtained in 5 microphotographs were added, and the percentage of positively stained investigated cells in mononuclear immune cell infiltrate was averaged. The ratios of examined T cell populations were calculated separately for their numbers in intratumoral area (intratumoral CTL/Treg number ratio) and at the invasion front (CTL/Treg number ratio at invasive margin), as well as for their percentages in immune cell infiltrate at invasion front (CTL/Treg percentage ratio at invasive margin).

### Statistical analysis

Distributions were tested for normality with Chi-square test. To assess the differences between groups, ANOVA Kruskal–Wallis and U Mann–Whitney tests were performed. A t-test was applied for normally distributed variables. The correlations between groups were evaluated by using Spearman rank test. Chi-square test was used to analyze associations between nominal variables. All analyses were performed using Statistica 13 (StatSoft Inc., USA). In brackets, the data were expressed as median (interquartile range); p values < 0.05 were considered statistically significant.

## Results

### Description of study group

The research group consisted of 87 cases of luminal A type BC. The average age of patients was 58.8 years (range, 32 to 88 years) at the time of diagnosis. Tumor size in 49 (56.32%) cases was of the pT1 stage and in 38 (43.68%) of the pT2 stage. The presence of lymphovascular invasion (LVI) was noted in 72 (82.76%) cases. Lymph nodes status as follows: pN0 in 29 (33.33%), pN1 in 36 (41.38%), pN2 in 11 (12.64%), and pN3 in 11 (12.64%) patients. In pN0 group, LVI was observed in 16 (55.17%) tumors and 13 (44.83%) cases lacked features of LVI. Regarding pTNM stage, stage I was diagnosed in 24 (27.59%) cases, II in 41 (47.13%) cases, and III in 22 (25.29%) cases. On the basis of the histologic type, 68 (78.16%) cases were classified as “not otherwise specified” (NOS), 16 (18.39%) cases as infiltrating lobular carcinoma (ILC), and 3 (3.45%) as “other.” Nottingham Histologic Grade was G1 in 29 (33.33%) cases, G2 in 47 (54.02%) cases, and G3 in 11 (12.65%) cases. The characteristics of patients and tumor data are summarized in Table 2.

**Table 2:** Clinicopathological features of the study group

Characteristic	Number of cases N = 87	%
Age [years]		
Range	32–88	
Mean	58.8	
Tumor size, n (%)		
pT1	49	56.3%
pT2	38	43.7%
Lymph nodes status, n(%)		
pN0	29	33.4%
pN1	36	41.4%
pN2	11	12.6%
pN3	11	12.6%
Lymphovascular invasion, n (%)		
Absent	15	17.2%
Present	72	82.8%
pTNM, n(%)		
I	24	27.6%
II	41	47.1%
III	22	25.3%
Nottingham Histologic Grade, n (%)		
G1	29	33.3%
G2	47	54.0%
G3	11	12.7%
Histologic type, n (%)		
NOS	68	78.2%
ILC	16	18.4%
Others	3	3.4%

### The relationships between nodal status of luminal A cancers and infiltrate of CTLs and Tregs

**Fig. 2:**
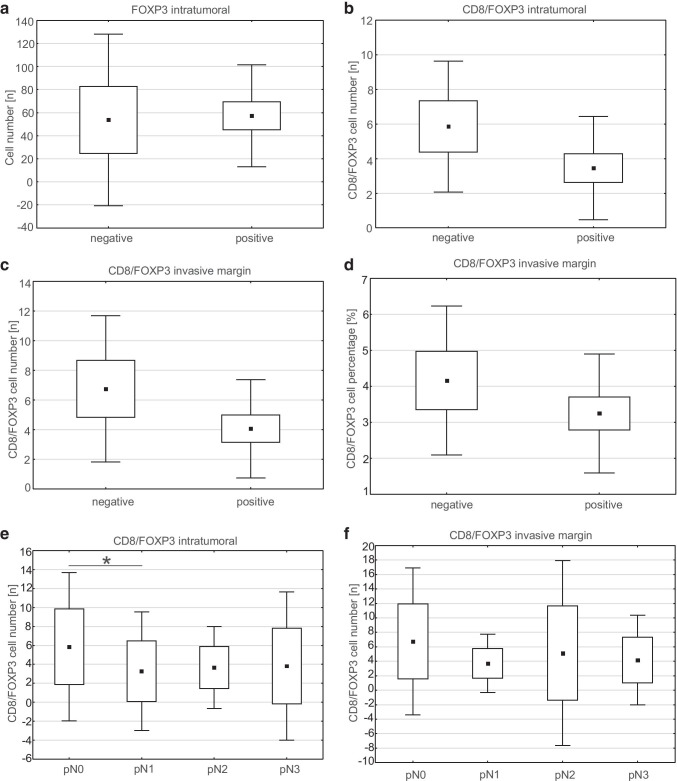
The differences and tendencies in CTL and Treg infiltrates with reference to nodal involvement. Relationships between nodal status (absence or presence of metastatic lymph nodes) and **A** number of intratumoral FOXP3 + Tregs (p = 0.029), **B** intratumoral CD8 + /FOXP3 + (CTL/Treg) cell number ratio (p = 0.001), **C** invasive margin CD8 + /FOXP3 + (CTL/Treg) cell number ratio (p = 0.009) and **D** invasive margin CD8 + /FOXP3 + (CTL/Treg) cell percentage ratio (p = 0.049). Relationships between pN stage and **E** intratumoral CD8 + /FOXP3 + (CTL/Treg) cell number ratio (p = 0.009) or **F** invasive margin CD8 + /FOXP3 + (CTL/Treg) cell number ratio (p = 0.08). A, B, C, D, U Mann–Whitney test: the central point is arithmetical mean, box is mean ± 2*standard error (SE), and whiskers are mean ± 0.95*standard deviation (SD); E, F, ANOVA Kruskal–Wallis test: the central point is arithmetical mean, box is mean ± SD, and whiskers are mean ± 1.96*SD. p-value < 0.05 was considered significant. *—p < 0.05 in post hoc test

To determine the associations between nodal involvement and infiltrate of CTLs and Tregs, we initially investigated the differences between node-negative and node-positive lesions. We noted that node-positive lesions showed significantly higher numbers of intratumoral Tregs as well as lower ratio of CTLs/Tregs in intratumoral area and at invasion margin (Fig. 2, Table 3).

**Table 3:** The association between infiltrate of CTLs and Tregs and prognostic factors in invasive breast cancer. Median (interquartile range)

		CTLs [n] intratumoral		CTLs [n] inv. margin		CTLs [%] inv. margin		Tregs [n] intratumoral		Tregs [n] inv. margin		Tregs [%] inv. margin		CTLs/Tregs [n] intratumoral		CTLs/Tregs [n] inv. margin		CTLs/Tregs [%] inv. margin	
		Median (quartiles)	p	Median (quartiles)	p	Median (quartiles)	p	Median (quartiles)	p	Median (quartiles)	p	Median (quartiles)	p	Median (quartiles)	p	Median (quartiles)	p	Median (quartiles)	p
LVI	Negative	122 (85–162)	0.38	348 (240–604)	0.88	35.50 (26.40–41.10)	0.54	19 (12–40)	**0.002**	45 (32–156)	**0.028**	8 (6–12)	**0.039**	5.31 (2.77–7.75)	**0.012**	5.73 (3.69–8.70)	**0.005**	3.83 (2.82–6.56)	**0.046**
	Positive	141 (60–257)		378 (249–594)		32.54 (24.90–39.80)		47.5 (25.5–70.5)		113 (63–228)		11 (8–14)		2.94 (1.64–4.55)		3.33 (1.89–5.42)		2.98 (1.96–4.00)	
Nodal status	Negative	132 (91–182)	0.69	417 (258–755)	0.15	39 (26.4–42.92)	0.11	21 (16–54)	**0.029**	84 (37–170)	0.23	9 (6–12)	0.15	4.55 (3.57–7)	**0.001**	5.31 (2.98–8.71)	**0.009**	3.43 (2.73–5.28)	**0.049**
	Positive	140 (58–257)		355 (222–492)		30.32 (25.7–38.8)		47 (30–70)		112 (58–228)		11 (8–14)		2.59 (1.32–3.99)		3.34 (1.87–4.97)		2.94 (1.96–3.99)	
Nodal stage	pN0	132 (91–182)	0.38	417 (258–755)	0.55	39 (26.4–42.92)	0.41	21 (16–54)	0.14	84 (37–170)	0.67	9 (6–12)	0.25	4.55 (3.57–7)	**0.009**	5.31 (2.98–8.7)	0.08	3.43 (2.73–5.28)	0.21
	pN1	132.5 (42.5–225)		359.5 (234–505)		29.5 (26.32–38.8)		47 (31–61.5)		112 (66–181)		11 (8.5–14)		2.41 (0.98–4.22)		3.37 (2.57–4.71)		2.93 (1.85–3.88)	
	pN2	229 (74–283)		378 (215–571)		32.54 (24.7–40.06)		70 (25–113)		133 (62–250)		14 (8–15.5)		3.88 (2.08–4.46)		3.03 (1.7–4.97)		2.86 (1.96–4.53)	
	pN3	148 (49–258)		331 (253–483)		33.85 (20.72–38.8)		45 (14–93)		106 (48–230)		9 (8–12)		2.52 (1.7–3.5)		2.54 (1.84–6.78)		3.2 (2.41–5.18)	
Tumor stage	pT1	134 (73.5–212)	0.77	365 (217–582.5)	0.87	30.19 (23.91–39.1)	0.20	40 (20–60)	0.24	86 (43–170)	0.10	10 (7–13)	0.09	3.9 (1.89–5.52)	0.26	4.09 (3.09–6.71)	0.051	3.08 (2.55–4.43)	0.58
	pT2	140.5 (58–257)		370.5 (260–676)		34.26 (28–41.5)		45.5 (33–93)		121.5 (74–230)		12 (8–16)		2.79 (1.7–4.46)		2.87 (1.89–4.97)		3.03 (1.91–4.47)	
Histologic grade	G1	100 (71–146)	**0.039**	320 (202–450)	0.13	28.2 (23.02–39.7)	0.39	32 (20–60)	0.54	84 (43–146)	**0.044**	9 (7–12)	0.07	2.84 (1.6–5.81)	0.73	4.31 (2.74–6.93)	0.40	2.86 (2.55–4.2)	0.54
	G2	144 (60–277)		356 (253–604)		35.3 (27.5–41.1)		21 (43–67)		116 (49–240)		12 (7–14)		3.5 (1.7–5.31)		3.47 (1.92–6.47)		3.22 (2.15–4.58)	
	G3	241.5 (157–258)		561.5 (400–732)		36.19 (30.32–39)		14 (54–95)		104 (93–372)		12 (9.5–22)		3.61 (2.94–3.99)		3.28 (2.15–4.33)		3.09 (1.38–3.81)	
Histologic type	NOS	144 (74–250)	0.26	393 (222–676)	0.35	32.64 (24.9–39.8)	0.67	43 (20.5–70.5)	0.72	112 (51–196)	0.85	11 (8–14)	0.13	3.5 (1.72–5.4)	0.51	3.38 (2.15–5.94)	0.98	2.94 (2.15–3.92)	0.19
	ILC	99 (59–160.5)		344 (253–422)		30.06 (26.04–39.45)		43 (35–55)		97 (48–203)		8.5 (6.25–12.5)		2.77 (1.38–4.19)		3.73 (2.07–7.42)		3.7 (2.35–5.23)	

With respect to pN stage, we noted its association with intratumoral CTL/Treg number ratio. In post hoc analysis, the significantly higher value was observed in pN0 than in pN1 tumors (p < 0.01). We found also similar tendency for CTL/Treg number ratio at invasion front (Fig. 2, Table 3). Interestingly, the mean values of both the above-mentioned ratios were slightly higher for pN2 and pN3 tumors as compared to pN1, but they did not exceed mean values observed in pN0 lesions (Fig. 2). We found that intratumoral and invasive margin CTL/Treg number ratios showed weak negative correlations (R =  − 0.25, p < 0.03, and R =  − 0.26, p < 0.02, respectively) with the number of metastatic lymph nodes.

We also investigated other stratifications of study group according to nodal involvement. When compared cases with ≤ 1 positive lymph node vs. ≥ 2 positive lymph nodes, the only difference concerned higher CTL/Treg number ratio at invasion front in cases with ≤ 1 positive lymph node [4.21 (2.74–6.93) vs. 3.21 (1.72–5.42), p < 0.05]. There were no statistically significant differences in infiltrate of analyzed cells between pN1 and pN > 1 as well as 1–2 and ≥ 3 metastatic lymph nodes (data not shown).

Regarding LVI, we noted that tumors with features of LVI showed significantly higher infiltrate of Tregs and lower CTL/Treg ratios (Table 3). In node-negative group, we observed that LVI-positive tumors had higher numbers of intratumoral and invasive margin Tregs than tumors without features of LVI [47.5 (19.50–111.0) vs. 19 (12–34), p < 0.03 in intratumoral area and 125 (71–234) vs. 45 (32–145), p < 0.05 at invasion front].

### The relationships between infiltrate of CTLs, Tregs, metastatic lymph nodes, and other prognostic markers in luminal A tumors

We observed that positive nodal status was associated with larger primary tumor diameter [21 (17–32) vs. 13 (11–21), p < 0.01] and lower PR expression [0.70 (0.50–0.80) vs. 0.85 (0.70–0.90), p < 0.02] in comparison to node-negative samples. LVI-positive tumors had positive nodal status more frequently than negative (77.8% vs. 22.2%, p < 0.001). Non-metastatic cases were more frequently of pT1 than pT2 stage (72.4% vs 27.6%, p < 0.04). No relationships between nodal involvement and patient age, histologic grade, mitotic index, ER, or Ki67 expression were found (data not shown).

Regarding tumor size, we found that pT2 tumors tended to have higher percentage of Tregs in infiltrate as well as lower CTL/Treg number ratio at invasion front (p = 0.087 and p = 0.051, respectively) than pT1 cancers. After the stratification for pT, in pT1 tumors, we observed positive correlations between the number of metastatic lymph nodes and the numbers of Tregs located intratumorally (R = 0.46, p < 0.01) and at invasive margin (R = 0.29, p < 0.05) as well as the percentage of Tregs (R = 0.35, p < 0.02) at invasion front. Opposite relationships were found for CTL/Treg number ratio in intratumoral and invasion front area (R =  − 0.37, p < 0.01 for both) as well for CTL/Treg percentage ratio at invasion front (R =  − 0.33, p < 0.03) in these cases. No significant differences were observed in pT2 group.

With regard to histologic grade, significantly greater number of intratumoral CTLs was observed in G3 than in G1 tumors (p < 0.05). Moreover, similar increase of Treg number and percentage with grade was noted at invasive edge of tumor (p < 0.05 and p = 0.068, respectively). No relationship was found with histologic type of tumor (Table 3).

The number of Tregs correlated negatively with PR expression both intratumorally and at invasive margin (R =  − 0.30, p < 0.01 and R =  − 0.29, p < 0.01, respectively). The same was noted for Treg population percentage in infiltrate at invasion front of tumor (R =  − 0.22, p < 0.04). The opposite association was observed for CTL/Treg number ratio at intratumoral and invasive edge area (R = 0.24, p < 0.03 and R = 0.32, p < 0.01). Mitotic index showed positive correlation with CTL number at intratumoral area (R = 0.38, p < 0.01) and invasion front (R = 0.39, p < 0.01) as well as with number of intratumoral and invasive margin Tregs (R = 0.29, p < 0.01 and R = 0.25, p < 0.02, respectively) and percentage of Tregs in mononuclear infiltrate located at invasion front (R = 0.30, p < 0.01). For Ki67 expression, its tendency for negative correlation with Treg percentage at tumor margin (R =  − 0.21, p < 0.06) was also identified.

The number of CTLs in intratumoral area and at invasive margin correlated positively with number and percentage of Tregs in both locations. Moreover, the percentage of CTLs in mononuclear infiltrate at invasion front correlated positively with all investigated CTL/Treg ratios. Conversely, the number and percentage of Tregs showed negative correlation with the values of CTL/Treg ratios (data not shown).

## Discussion

As immunohistochemistry-based identification of BC molecular subtypes is imperfect, great efforts have been made to stratify luminal A BC subtype according to progression risk and treatment options [7, 19]. Based on micro-RNA expression study, two luminal A subtypes, which differ in clinical outcome, were identified. High expression of stromal cell–derived proteins in luminal A BC were associated with better prognosis [19]. Guo et al. observed that microenvironment of high-risk luminal A subgroup is characterized by impaired immune response [20]. Some authors postulate that distinct gene expression pattern as well as certain gene mutations underlies biological differences between subtypes and subsequently affect varied immune cell infiltration [21, 22].

Due to significantly lower rates of immunological markers and modest immune infiltrate as compared to other subtypes, luminal A BC is considered to be non-immunogenic [5, 9, 21, 23, 24]. Thus, the vast majority of research investigating immune microenvironment in BC focus on its non-luminal types. Chung et al. suggested that aggressive ER-negative cancers show high genomic instability that attracts TILs. TILs, in turn, stimulate tumor cells to express antigen-presenting molecules [25]. Conversely, higher TILs density in these tumors were linked to higher human leukocyte antigen class I (HLA I) expression induced by lymphocyte-independent, autonomous interferon (IFN) signaling in cancer cells [23, 25] and to distinct level of sialylation of several cell membrane proteins in HR-negative BC [26]. The increase in the expression of TILs, CTLs, and Tregs in mammary tissue during the progression of non-luminal cancer is well-documented [24, 27–29]. The presence of CTLs and Tregs appears to be associated with the expression of HLA I in cancer tissue, which is low in ER-positive BC [23, 25]. There are data indicating that the numbers of CTLs and Tregs are significantly decreased in luminal A in comparison to non-luminal cancers [21, 26, 29–32]. These observations were confirmed by our previous study [9]. Some authors postulate that in ER-positive tumors, higher TIL density is associated with more aggressive course [10] and worse prognosis, which is explained by the concurrent absence of CTLs within tumor islets [22]. However, the prognostic impact of CTLs and Tregs on BC and, in particular, its ER-positive subtypes, remains controversial [11, 30, 33, 34]. Our previous research revealed that in luminal cancers, more numerous CTLs and Tregs are related to greater tumor diameter [9].

Cancer spread to lymph nodes is one of the most important prognostic factors in BC [8, 34]; thus, the association between TILs and nodal stage is an interesting issue to explore. Some authors postulate that tumor microenvironment generates immunosuppressive niche in lymph nodes that facilitates the formation of metastases [3], while others question such a relationship [29]. According to the results of some authors, strong HLA I expression appears to be a protective factor against nodal spread of BC [23, 25]. The association between CTLs or Tregs and nodal status in BC has not been fully elucidated. It was observed that both the decreased CTLs and the increased Tregs in early stage BC are associated with lymph node metastases [32, 35]. On the other hand, Sheu et al. noted more prevalent CTLs in the infiltrate of lymph node-positive BCs [27]. There were also studies showing no relationship between the two T cell types and nodal stage [21, 30, 36]. Our previous study implicated that non-luminal and luminal BCs show different pattern of CTL and Treg infiltrate with regard to nodal involvement [9].

The information on CTL infiltrate in luminal BC is scarce. Data obtained from ER-negative subtypes indicate, on the one side, the role of these cells in spontaneous healing [37] and favorable prognosis [37, 38] and, on the other, an inactive, exhaustive state of CTLs, which supports immune escape [30, 34, 39]. The few studies conducted on luminal tumors show higher CTL percentage in these subtypes than in others, which is associated with better survival [29, 40]. This phenomenon can be explained by the existence of antigen-specific CTLs, which are able to eliminate cancer stem cells [41]. In a study by Cimino-Matthews et al., CTLs were the only cell population that was decreased in metastatic tumors in comparison to primary lesions in luminal cancers [31]. The authors noted that denser CTL infiltrate was related to poor tumor differentiation and higher mitotic index that is the factors of worse prognosis. This is consistent with our results.

Tregs impair anti-tumoral function of other lymphocytes [29]. Prognostic significance and function of tumor-infiltrating Tregs appears to vary across different malignancies, possibly due to varied interaction between these cells, microenvironment, and signaling pathways [13]. In BC, high levels of Tregs are associated with low levels of CTL-derived cytokines [29], increased expression of indoleamine 2,3-dioxygenase (IDO), which is regarded as Treg inductive factor [32, 42], and hypoxia that contributes to their recruitment, particularly in clinically aggressive subtypes [43]. Pronounced Treg infiltrate is related to high grade, HER2-positivity, basal-like phenotype [13, 43], and poor patient survival [32, 42]. Some authors postulate that at least some of these relationships are independent of ER status [13, 28, 30, 43]. In non-luminal cancers, Tregs are supposed to induce immune tolerance [30]. The increase in the numbers of Tregs in BC tumor tissue during its spread indicates the enhancement of immune suppression in this process [31], but their relationship with nodal involvement has not been established yet [30]. In the present study, we observed that denser intratumoral Treg infiltrate is associated with positive nodal status and higher pT, suggesting that Treg influx into tumor islets is related to the progression of luminal A BC. Moreover, correlations between dense Treg infiltrate and higher grade, mitotic index, or lower PR indicate their relationship with more aggressive cancer features.

The balance between CTLs and Tregs reflects a shift in immune response — the predominance of cytotoxic over regulatory cells, which is an indicator of effective immune response [31] or the prevalence of Tregs, which suggests immune suppression [21]. The expression of HLA I, a tumor grade, and the proliferation rate are factors that affect CTL/Treg ratio [25]. High CTL/Treg ratio in primary BC tissue is associated with improved survival [25, 30, 31, 38] and lower recurrence rates, suggesting that the activation of cytotoxic functions of immune system prevents the formation of metastases [38]. This appears to be consistent with our results, as higher CTL/Treg ratio was observed in primary luminal A lesions without nodal involvement and of smaller diameter. In terms of pN, it is noteworthy that the only significant difference in CTL/Treg ratio was found between pN0 and pN1 tumors and no difference was noted between pN1 and pN2 cancers. Moreover, adverse correlation between the number of metastatic lymph nodes and CTL/Treg ratio and positive correlation between Treg density and metastatic lymph nodes number were observed in small-sized (pT1) tumors. Therefore, we hypothesize that the role of interplay between tumor-infiltrating CTLs and Tregs in nodal spread is the most important in the initial phase of BC progression and rather results from change in Treg density than CTL decrease. In a few research studies, luminal tumors presented with higher CTL/Treg ratio than other BC subtypes [21, 30].

LVI observed in peritumoral area of pN0 primary BC is a prognostic factor strongly related to lymph node metastases [6, 36]. Although relationship between LVI and TIL subpopulation in BC is debatable [21, 25, 27, 36], we found that higher numbers of Tregs were related to LVI in node-negative luminal A BC. Again, this would suggest that Tregs are pivotal contributors to the initiation of lymph node metastases. Moreover, in our study, the presence of lymph node metastases correlates with larger primary tumor size in comparison to tumors with the pN0 feature. In addition, tumors that did not metastasize to lymph nodes showed more frequently pT1 stage than pT2. The similar association was noted by Pehlivan et al. [36]. Van Calster et al. also showed that the increase in tumor diameter of 1 cm significantly increases the risk of lymph node metastasis, but these data did not include the molecular classification of breast cancer subtypes [44]. The associations between pN stage and both tumor size and T stage were shown in ER-positive/HER2-negative BC by Noda et al. [33]. Several other authors have obtained similar results [45], but some did not show any relationships between tumor size and lymph node involvement in luminal A BC [46]. Our study showed that patient age does not correlate with lymph node involvement in this molecular subtype. These results are confirmed by other authors [33, 46, 47]. Surprisingly, Van Calster et al. showed that the increase of patient age by 10 years reduces the chance of nodal involvement [44].

Our observations show that lymph node involvement is not dependent on the degree of tumor differentiation. Our findings confirm the observations of Tullberg et al. concerning luminal A cancer [46] as well as studies conducted in breast cancers regardless of their molecular subtype [47]. On the contrary, Van Calster et al. showed that tumor grade correlates with lymph node involvement in all subtypes of breast cancer [44]. Moreover, we demonstrated that an increase in tumor lymph node involvement inversely correlates with PR expression. Many authors did not confirm this observations [33, 44, 47]. However, these studies concerned all subtypes of breast cancer, including those in which the expression of PR is assumed to be low. Thus, we hypothesize that decreased lymph node involvement is relevant only in cases with significant expression of PR. This observation supports the view that high PR level is an indicator of good prognosis in luminal A cancer. In line with other studies, our findings showed that there is no correlation between lymph node involvement and ER expression [44, 47].

We showed that mitotic index as well as Ki-67 expression did not change with an increase in pN stage. Liikanen et al. obtained similar results [47]. In contrast, Rossi et al. showed that in patients with high mitotic index and high Ki67, lymph node metastases were more prevalent compared to those with low mitotic index. However, in this study, T3 tumors were considered, without the stratification into molecular subtypes of BC [48]. Considering our above-mentioned findings, we hypothesize that in luminal A BC, lymphovascular spread develops with tumor growth and does not results from its more aggressive phenotype.

The results of this study indicate that denser intratumoral Treg infiltrate and lower CTL/Treg ratio are associated with LVI, positive lymph nodes and more numerous metastatic lymph nodes in luminal A BC. Moreover, more numerous Tregs and lower CTL/Treg ratio are related to greater tumor diameter, higher histologic grade, lower PR expression, and higher proliferation rate. On the other hand, denser tumor-associated CTL infiltrate is observed in high grade and more proliferating tumors. This suggests that Treg and CTL infiltrates are implicated in the initial phase of progression and spread of luminal A BC. With regard to other prognostic factors in this subtype, only tumor size and lower PR expression showed associations with nodal involvement. Therefore, we hypothesize that the levels of tumor-infiltrating regulatory and cytotoxic cells, the balance between them, and the interplay between T cell infiltrate and other conventional clinicopathological indicators contribute to nodal spread of luminal A BC.

## Data Availability

Not applicable.
